# Efficient simulation of the spatial transmission dynamics of influenza

**DOI:** 10.1371/currents.RRN1141

**Published:** 2010-02-01

**Authors:** Meng-Tsung Tsai, Tsurng-Chen Chern, Jen-Hsiang Chuang, Chih-Wen Hsueh, Hsu-Sung Kuo, Churn-Jung Liau, Steven Riley, Bing-Jie Shen, Da-Wei Wang, Chih-hao Shen, Tsan-sheng Hsu (corresponding author)

**Affiliations:** ^*^Research Assistant at Institute of Information Science, Academia Sinica, Taipei, Taiwan; ^†^Institute of Information Science, Academia Sinica, Taipei, Taiwan; ^‡^Centers for Disease Control Taiwan; ^§^National Taiwan University, Dept of CSIE; ^¶^TECRO; ^#^Researcher at Institute of Information Science, Academia Sinica, Taipei, Taiwan; ^**^The University of Hong Kong; ^††^Division of Radiation Oncology, Department of Radiation, Far Eastern Memorial Hospital; ^‡‡^Institute of Information Science, Academia Sinica and ^§§^University of Virginia

## Abstract

Early data from the 2009 H1N1 pandemic (H1N1pdm) suggest that previous studies over-estimated the within-country rate of spatial spread of pandemic influenza. As large spatially-resolved data sets are constructed, the need for efficient simulation code with which to investigate the spatial patterns of the pandemic becomes clear. Here, we describe a significant improvement in the efficiency of an individual-based stochastic disease simulation framework that has been used for multiple previous studies. We quantify the efficiency of the revised algorithm and present an alternative parameterization of the model in terms of the basic reproductive number. We apply the model to the population of Taiwan and demonstrate how the location of the initial seed can influence spatial incidence profiles and the overall spread of the epidemic. Differences in incidence are driven by the relative connectivity of alternate seed locations.

## Introduction 

The current global spread of a novel influenza strain [1] highlights gaps in our understanding of the spatial component of disease transmission at national and regional scales. For example, the early summer 2009 wave in the United States has affected some populations much more so than others (Centers for Disease Control, USA), even populations at similar latitudes. Also, there was substantial transmission in parts of southern England throughout the summer 2009, but very little in most of northern mainland Europe (European Centre for Disease Prevention and Control). This slow progression to national and regional level synchrony is not obviously consistent with previous theoretical studies of the within-country dynamics of pandemic influenza [2] [3] [4], in which census-reported commuting patterns and airline flight data were used to characterize very rapid spatial spread. Explaining these early patterns of spatial spread for the 2009 pandemic will likely be an active area of epidemiological research in the coming years.

Stochastic spatial transmission models, in which individuals or small communities are represented explicitly in space, are an extension of more traditional approaches and have been a valuable tool in the study of infectious diseases in humans and animals [5]. Traditionally, mathematical models of epidemics often take the form of deterministic differential equations in which the variables represent the expected number of individuals in broad disease classes (e.g. susceptible, infected, or recovered) [6]. Although such models can be extended to model the geographic spread of infectious diseases on patches [7], when it is not clear which spatial scales are most important, it is difficult to use compartmental approaches with confidence. 

Here, we describe an algorithmic refinement of a spatial stochastic model of individuals and their communities. This framework was originally designed to investigate community interventions against influenza in a generic sense [8]. It was later extended to examine the optimal response to a bio-terrorist smallpox attack [9] and also to examine the potential for the containment if an influenza pandemic in large well mixed population [10]. A spatial component was added to the model to study the feasibility of containing an emergent influenza pandemic in a rural setting in southeast Asia [11]. In its last major development, the underlying algorithm was parallelized to allow it to run with a population of 300 million and used to predict the likely impact of mitigation measures on an influenza pandemic in the United States [2]. More recently, the same framework has been used to describe the likely fall wave transmission dynamics for H1N1pdm in Los Angeles county [12].

We have implemented a more efficient algorithm for this popular disease transmission model. We demonstrate increased computational efficiency compared with previous implementations and we describe a parameterization scheme for the model using the basic reproductive number, rather than the per contact transmission potential. We illustrate the utility of the refined model with simulation studies of seeding dynamics for a pandemic of influenza in Taiwan.  

## Methods 

The model is, effectively, a highly connected network model. Individuals are fully connected within different settings (home, school, workplace, etc) and we assume that all members of one instance of a setting are connected to all other members. The infection process over this network can be though of in two stages: contact and infection. Each individual is a member of one of five age groups and has several contact groups. The age groups are preschoolers (0 - 4 years), school-age children (5 -18 years), young adults (19 - 29 years), adults (30 - 64 years), and elders (65+ years). The social contact groups include communities, neighborhoods, household clusters, households, work groups, high schools, middle schools, elementary schools, daycare centers, and play groups. Individuals have contacts in their contact groups every day. Each day is divided into a daytime and nighttime period. During nighttime, contacts occur only in communities, neighborhoods, household clusters, and households; whereas in the daytime, contacts occur in all the contact groups. Each contact between an infected and a susceptible individuals results in a potential infection. The probability that an infected individual can transmit the virus to a susceptible individual is called the contact probability and depends on the group in which they have contacts and the ages of the potential infector and infectee. The age of an individual not only affects the probability that an individual is infected, it also determines an individual's daytime contact groups: preschoolers stay either in daycare centers or in play groups; school-age children stay either in schools or in households as drop-outs; and young adults and adults stay either in work groups or in households if unemployed. Further details of the basic model can be found in the supporting information to Ref [2].  

The discrete-time simulation of infection events in individual-based epidemic models can be reduced to the generation of binomial deviates. Within any given model there can be many types of infectious individual and many types of susceptible individual. For example, there can be many age groups and many stages in the natural history of the disease. The set of all possible pairs in which the first element is an infectious individual and the other element is a susceptible individual (an *I*-*S* pair) defines the set from which infection events can be simulated at any point in time. If many of the pairs have exactly the same probability of generating an infection (*S *of exactly same type and *I *of the exactly the same type) then many infection events can be generated with relatively few binomial deviates. However, if the pairs are largely different, then many binomial deviates need to be drawn to generate a similar number of infections. The introduction of spatial dimensions into individual-based formulations greatly increases the heterogeneity of the model because every small group of individuals with a unique location forms, effectively, their own risk group. 

A high-level description of a naive algorithm for the basic model is as follows:

**foreach** time period *T* **do**     **foreach** infected individual *I* **do**       **begin**       (1) update the status of* I *according to *T*       (2) **if** *I *is a possible infector **then**                **begin**                    **foreach** individual* S* **do**                         **if** *S* is a susceptible individual **then**                              **foreach** possible contact group *G* **do**                                 **if** *I *and *S* are in the same group *G* **then                                    begin**

(1) calculate the probability* p_Is_, *that *S* is infected by* I  *  (2) use a random number generator to decide whether *S *is infected by* I *for a probability of* p* *_Is_* (3) update the status of *S *if *S* is infected 

                                  **end**                 **end**        **end**

The algorithm we have developed greatly improves the efficiency with which infection events can be generated across large numbers of similar risk pairs. Here, we briefly describe the key features of the algorithm as it relates to the efficient simulation of spatial epidemics. The methods are described in more detail elsewhere [13]. In essence, the approach is to use lazy evaluation for large groups of pairs with similar probabilities of an infection event. For example, one infectious individual *a* in community *A* has a certain maximum probability of infecting members of community *B*, based on the flow of workers between those two communities. The precise probability of infection for each member of community *B* will depend on their age and other risk variables. However, the maximum probability for any individual in group *B*, *p*_*max*_, may be very small if the worker flow between *A* and *B* is small. Therefore, using the Sieve algorithm, our first step is to generate a random variable for the provisional number of infection events that occur by assuming that all pairs have the same probability of an infection occurring. This generates too many infections. The second step is to select specific pairs at random and either accept or reject provisional infections using the precise probability of infection between individual *a* and each individual *b* (in the provisional set of infections in community *B*). We define the precise probability to be *p*_*b*_. If we accept each provisional infection event with probability  *p*_*b*_ / *p*_*max*_, it is clear that the overall probability of individual b being infected is equal to *p*_*b *_. Therefore, our method reproduces the same stochastic process as if we evaluated each individual  *p*_*b *_ separately and is not an approximation. 

A high-level description of our improved algorithm is as follows:

**foreach **time period *T* **do**     **foreach **infected individual *I* **do**     ** begin**         (1) update the status of *I *according to* T*         (2) **foreach **possible contact group* G *that *I *is in **do**                **begin**

(1) calculate the possible infection probabilities *p_Is_* between *I* and all susceptible individuals *S *within *G* (2) use the Sieve algorithm below to decide all of the individuals within *G* to be infected by the individual* I* (3) update the status of newly infected individuals 

                **end**

     **end**

The Sieve algorithm  

(1) let *p*_*max*_ = max{*p* *_Is_*} for all susceptible individual *S* in *G*

(2) let *N* be the number of susceptible individuals in *G* (3) first decide a tight bound *K *which is the upper bound of possible infected persons according to a binomial distribution with an inclusion probability of *p*_*max*_ and *N *trials (4) randomly pick* K* candidates from the group of susceptible individuals in *G* (5) **foreach** picked candidate *b* **do**

use a random number generator to decide whether *b* will be infected according with a probability *p_Ib_/* *p*_*max*_

We are able to prove the statistical behaviors of the Sieve algorithm are the same with the naive algorithm where each candidate is decided one by once sequential. The proof of equivalence is given in [13]. Note that our Sieve algorithm decides a set of candidates in a batch. One of the reasons that our algorithm can run faster is because that  in  practice *p*_*max  *_is very small. Thus the size of the candidates *K *selected in the Sieve algorithm is much small than *N *which is the pool of people to be considered. 

By treating the model explicitly as a network, we calculate the average number of secondary cases *a priori*, rather than using semi-empirical methods to calibrate the model. The basic reproductive number *R*_*0*_ is the expected number of secondary infections generated by a single typically infectious individual in an otherwise susceptible population [14].  *R*_*0*_ is a threshold parameter that determines whether or not an infectious disease will spread through a population. Strictly, for models with multiple types of infectious individuals, *R*_*0*_ should be defined in terms of a next generation matrix and an eigen vector for the exponential phase of growth. The eigen vector is important in that it defines what is typical during the exponential phase. Often, a typical type of infectious individual will be different from a randomly chosen individual. For network models of infectious disease, the formal approach presents some problems because every individual is, essentially, a different type. Therefore, we follow many previous network models and use the average number of secondary cases per randomly chosen individual as  *R*_*0*_. 

Based on the influenza model and parameters, we compute the probability that infectious individual *i* infects susceptible individual *j*, namely *w*_*ij*_ as follows. First, the infection probability resulting from *i* and *j*'s contact in group *k* is defined to be *p_ijk_*= *P_trans_*multiplies* c_k_*, where *P*_*trans*_ is the disease-dependent transmission probability and *c_k_* is the group-dependent contact probability. Second, *D_*ij*_* is the set of *i* and *j*'s contact groups in the daytime, and *N_ij_* is the set of *i* and *j*'s contact groups during nighttime. The intersection of *D_ij_* and *N_ij_* can be either empty or nonempty. Third, when the infectious individuals are incubating or asymptomatic, the infection probability is reduced by a factor, *r*, where *r* > 1. For clarity, we define  *h_ijk_* =  *p_ijk_*_* *_/ *r*. In our model, the current setting of *r* is set to 2 following  Ref [2]. Finally, by taking all daytime and nighttime contacts into consideration, the daily infection probability is calculated by

\begin{equation*}\[ P_{ij} = 1-\left[\mbox{$\prod_{k\in D_{ij}}$}(1-p_{ijk})\right]\left[\mbox{$\prod_{k\in N_{ij}}$}(1-p_{ijk})\right], \]\end{equation*}

\begin{equation*}\[ H_{ij} = 1-\left[\mbox{$\prod_{k\in D_{ij}}$}(1-h_{ijk})\right]\left[\mbox{$\prod_{k\in N_{ij}}$}(1-h_{ijk})\right], \]\end{equation*}

where *H*_*ij*_ is the daily infection probability when individual *i* is incubating or asymptomatic. By adopting the natural history model, the computation of the total expected number of infections can be calculated (Figure 1). Also, we validated the accuracy of the calculation by simulation (Table 1). 

**Figure:**
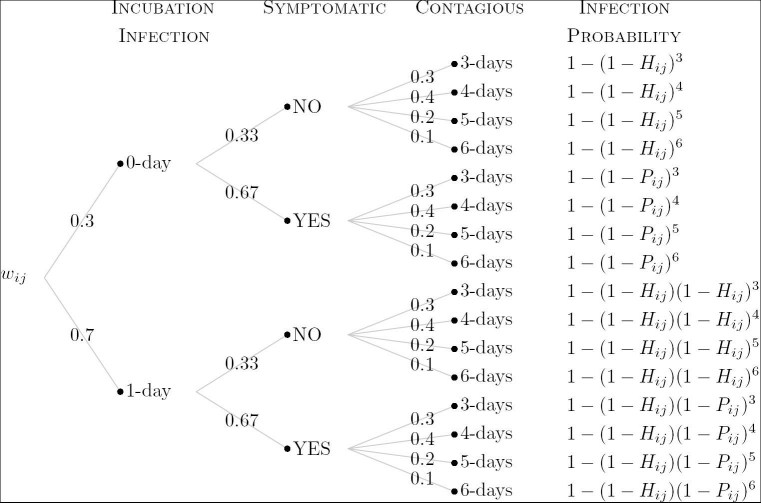
**Figure 1.** The computation of the probability that individual* j *is infected by individual* i *according to the natural history model. 

**Figure:**
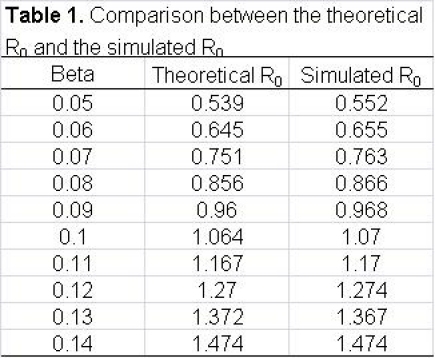

## Results

The Sieve algorithm was substantially more efficient when applied to a real-world application than the naive algorithm. We used a population of 23 million people (approximately equal to the size of Taiwan's population) as our example. We calibrated the strength of transmission to have a cumulative infection attack of 60% (a severe pandemic) and let the duration of infectiousness be, on average, 3 days. Even with a coarse 0.5 day time step, the naive algorithm would still require of order 10^15^ interactions to be evaluated (if we assume every infectious individual has a non-zero probability of infecting every susceptible individual). By using the re-sampling approach in the Sieve algorithm, the efficiency is dramatically improved with no loss of precision (Figure 2a).

**Figure:**
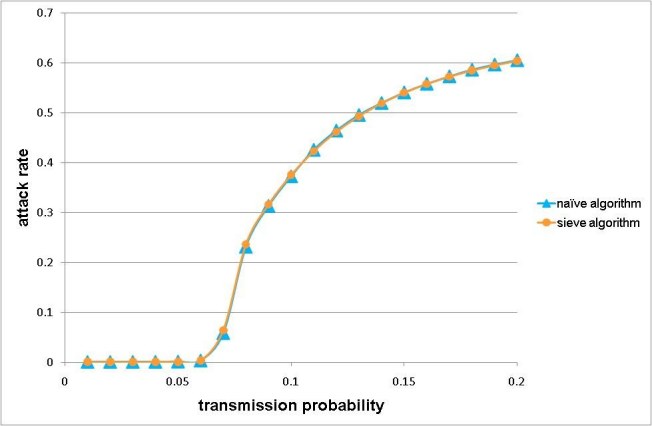

**Figure:**
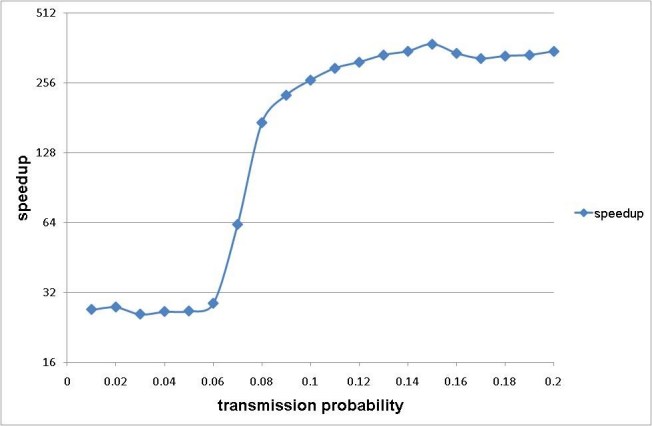

**Figure 2.** The precision and efficiency of the Sieve algorithm, as applied to a model of pandemic influenza transmission in Taiwan. a) Demonstrates the correct implementation of the algorithm in that the attack rate from the Sieve algorithm is the same as the attack rate from the naive algorithm for a range of values of transmissibility. b) Shows the speedup of the Sieve algorithm for a range of values of transmissibility. Speedup is defined as the ratio of the average computation time for the naive algorithm over that of the Sieve algorithm. 

The improved efficiency allows rapid simulation of pandemic influenza outbreaks at the national scale for Taiwan. On a machine with AMD Opteron OSA250, dual-core, 2.4 GHz CPU and 4GB DDR1 memory, our implementation completed one simulation in less than 5 minutes for a wide range of values of transmissibility. Simulation time was short up to a threshold value after which the cumulative number of infections increased substantially. The peak in simulation time does not correspond to the peak in numbers of infections, which is to the far right of the chart. The peak simulation time occurs at a lower cumulative attack rates because there is a trade off between the duration of the epidemic and the total number of infections. Large epidemics are faster. When the epidemic is very quick, there are far fewer time steps in which many infections occur. However, when the epidemic is a little smaller but much slower, the computational burden is at its highest.

Increased efficiency allows us to conduct simulations many times rather than being forced to report runs from a single realization. Therefore, we conducted a number of experiments to assess the degree of variability introduced by the process of generating the mock population and by the simulation algorithm itself. 

First, we consider the variation introduced when, given different seeds but the same demographic data, the Initialization module generates many different mock populations. We carried out the following experiment: we randomly picked a mock population and simulated 2,000 baseline realizations constant transmission parameters. For each of the 2,000 realizations, we extracted the average day of 1000th symptomatic case and the average total number of infections. We then treated the statistics from all 2,000 results as if they are the real sample space and assume that the parameters of the real unknown sample space is not too far away from it. Now each production run is merely a sample from the 2000-run sample space. We first observe that the histograms of those important properties are all bell-shaped. We use maximum likelihood heuristic to estimate the most likely normal distribution to match the histogram. In Figure 3, the histogram and the estimated normal distribution are shown on the left. We then compare observed distribution with the theoretical normal distribution in quantile-quantile (q-q) plot. The q-q plot is a graphical technique for determining if two data sets come from populations with a common distribution. It is a plot of the quantiles of the first data set against the quantiles of the second data set. By a quantile, we mean the fraction (or percent) of points below the given value. A 45-degree reference line is also plotted. If the two sets come from a population with the same distribution, the points should fall approximately along this reference line [15]. As we can see, the normality of observed distribution is not only visually correlated on the left but also statistically verifiable on the right.   

**Figure:**
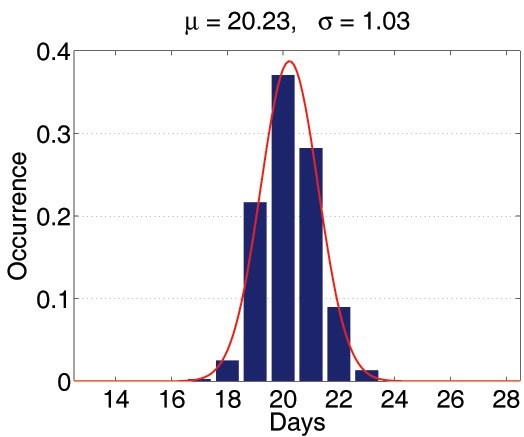

**Figure:**
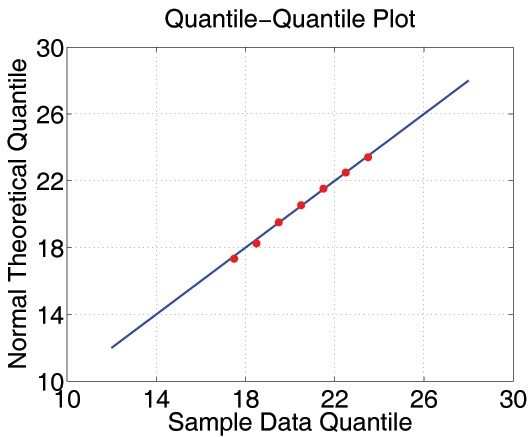

**Figure 3.** The statistical properties of simulations with different mock populations. 

Second, we study the relationship between the width of the confidence interval and the number of realizations. In Table 2, we summarize the width of confidence interval of several important measurements for sample size equal to 20, 30 and 40. Based on these numbers, it is safe to say that a sensible decision is to repeat each simulation 30 times.

**Figure:**
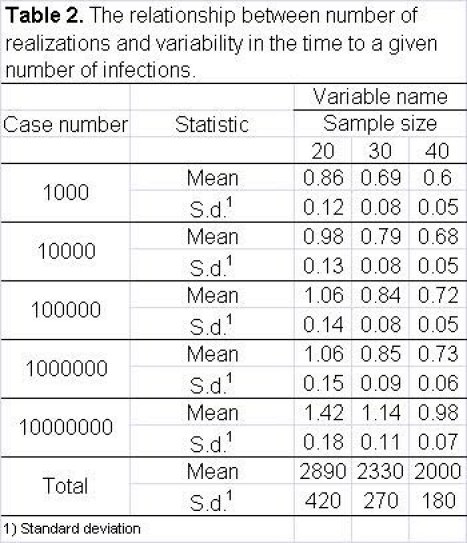

 We illustrate the practical use of the model by simulating severe pandemics in Taiwan. Movie 1 demonstrates the comparison of two baseline scenarios. From the movie, a number of general aspects of the spatial epidemiology of infectious disease can be observed. First, the capital city, Taipei, is better connected than the mid-latitude city of Chuanghua. The epidemic progressed more rapidly from Taipei to other areas, resulting in a more synchronized epidemic: incidence is at similar values in quite distant locations during the middle part of the epidemic. In contrast, Chuanghua is less well connected and the epidemic seeded there takes longer to spread to other parts of the population. Hence, incidence in the mid-latitudes close to the seed leads incidence in other areas. This results in a slightly faster and 'less peaky' epidemic. These simulation runs illustrate the general principal that when epidemics fail to synchronize spatially, overall incidence is less peaked. However, the result presented here do not describe local incidence of infection, which will be more 'peaky'.   

See "Taiwan Influenza Epidemic Simulations".

## Discussion

 We have described the application of a general re-sampling algorithm to a widely used spatial model of infectious disease transmission [8]. The resulting epidemic simulation tool achieves substantial speedups compared with our own implementation of a naive algorithm for the same model. Although derived independently, the resulting simulation algorithm is similar to those used to investigate the properties of the re-emergence of smallpox in the UK [16], and the pandemic influenza in Thailand [17], the United Kingdom and the United States [4].

We suggest that further research on the underlying algorithms for the model presented here and other similar models is warranted. For example, there are many ecological questions about the spatial properties of the current H1N1 pandemic -- not least the need to explain the high degree of spatio-temporal variability observed at the continental scale. More generally, at any scale, improved computational efficiency of epidemic models, similar to that demonstrated here, will substantially increase their utility as tools for theoretical investigation.   

## Appendix: Implementation details

To construct an efficient influenza simulation system aiming at Taiwan population, we base our model on Taiwan regional geographic data, Taiwan Census 2000 data (http://www.stat.gov.tw) and develop an integrated three modules, Initialization, Simulation, and Visualization, system as briefly shown in Figure A1. With the adaptation of various techniques in software optimization, not only is our system capable of evaluating containment strategies against influenza, the efficiency of our system also makes it possible to perform multiple courses, within reason, of the same simulation over and over again. The introduction to components of our system is as follows:

**Figure:**
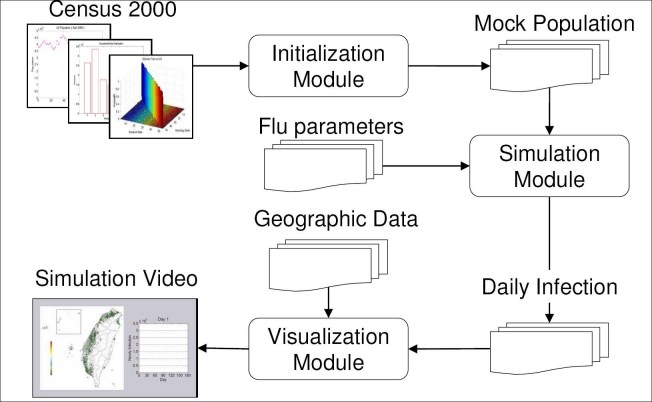
**Figure A1.** System architecture.

****

**Initialization module.** This module first reads a number of configuration files: *Ps* lists the town's id with the number of communities under this township, 369 entries for 369 towns in Taiwan; *age.tbl* lists the age and the number of people of that age, 101 entries for age 0 to 100, people with age 100+ years old are counted as age 100; *hst.tbl* lists the family structures and the number of families of that structure, the family structure is defined as a five digit number, with the first digit (most significant digit) denotes the number of elders (65+ years old) living in that family, the second digits for the number of adults (30 - 64 years old), and so forth, please also note that the sum of all 5 digits will not exceed 7, since each household consists of one to seven people; *wflow.tbl* is the 369 by 369 matrix depicts the town-to-town worker flow as described in the previous paragraph. The Initialization module will then go through all the towns, create communities for each town, create neighborhoods for each community, as well as the contact groups for each neighborhood, or community depends on the time period and activity patterns. During this process, each household is also created stochastically to follow family structure distribution information from *hst.tbl*, people are populated into each household according to the age groups as defined by the family structure of that household. The exact age of each person is also selected stochastically based on information from *age.tbl*. Now that the basic model of Taiwan population is generated with individual's age and residence information, we'll go through all the people and assigned them to appropriate contact groups according to their age and social activity patterns pertaining to Taiwanese way of living. For example, with working adults (age 19 - 64), we consider 5% of unemployment rate and assign the unemployed individuals to stay at home during the day period, and others to a work group with location stochastically selected based on information from *wflow.tbl*. We also consider 7% of drop-out rate for school age from 5 to 18. One final step is to adjust the size of each group if necessary. It is worth mentioning that the module takes less than a minute to generate a mock Taiwan population of 23 million people.

**Mock population. **Once the Initialization module generates a mock population, the social network (daily contact patterns) among individuals is fixed. Each individual belongs to one out of five age groups, in the mean time, he/she belongs to multiple contact groups according to his/her daytime and nighttime activities. To speed up the simulation, we pay special attention to memory access patterns so that a high hitting rate of cache memory can be maintained. The results of repeated and complicated floating point computations are stored to avoid recomputing. The status of communities and contact groups, as well as the attributes which reflect each individual's current state are carefully arranged to reduce memory consumption and access time. Grounded on such a computational social network model, the Simulation module is able to simulate the influenza epidemic effectively and efficiently.

**Simulation module.** Taking the mock population and influenza parameters as inputs, this module simulates the epidemic progression in Taiwan. We had designed several ways to introduce index cases to initiate the epidemic, one of the methods is that we assume international air travelers are the dominant source of influenza infectors, and we can proportionally seed index cases according to the travel patterns and the prevalence of the epidemic regions. In the accompanied examples, five individuals are chosen at random as the infectors at the first day of the simulation, they are seeded in (1) the heart of Taipei city, a popular shopping district which is the first stop of the majority of foreign and domestic tourists, (2) a small farming township in Changhua county, where they have the highest concentration of chicken livestock in the country. Both simulations cover 180 days, roughly the length of a Taiwan as well as U.S. influenza season. In the Simulation module, we neglect to implement the occasional long distance travel as stated in the supporting information to Ref [2]. The reason is that Taiwan is a relatively small island, about 400 km long and 145 km wide. The distance of those occasional travel, be it for business or leisure, is most likely not going to exceed the distance covered by the daily commuter travel for work. The worker flow data should be the dominant force of spreading the disease. 

The module runs in cycles of two 12-hour periods (daytime and nighttime), during which we identify several contact groups. In five of these groups (households, household clusters, preschools, play groups, schools, and work groups), relatively close contact regularly occurs. Additionally, neighborhoods and communities are for modeling unspecified groups, e.g. shopping malls, within which occasional contact occurs. Daytime contact occurs in all contact groups, while nighttime contact occurs only in households, household clusters, neighborhoods, and communities.

Conceptually, for each contact group we calculate the infection probability for its susceptible members according to the infectors within. After running through all contact groups, we flip a coin to decide if the susceptible individual gets infected. Following the influenza model introduced in Section 2, influenza parameters from [2], and the person-to-person contacts shown in the mock population, the Simulation module outputs the number of newly infected individuals after every 24-hour simulation.

**Long distance trips.** We did not implement the long distance air trip because the worker flow data capture the daily activity well and Taiwan is a relatively small island with limited air-traveling. 

**Visualization module.** In order to make the results of the Simulation module self-explanatory, the Visualization module transforms these seemingly meaningless numbers into a motion picture, which records the daily new cases for each local government as the influenza epidemic progresses. The color of the dot varies from green to red indicating the severity of the flu epidemic from safe to critical.

### **Funding Information**

This work was supported in part by the following grants: DOH98-DC-2036 from the Centers for Disease Control, Department of Health, Taiwan, R.O.C.; 97-2221-E-001-011-MY3 from National Science Council, Taiwan, R.O.C.; 98-2221-E-001-013-MY3 from National Science Council, Taiwan, R.O.C.; R01 TW008246-01 from Fogerty International Centre; RAPIDD program from Fogerty International Centre with the Science&Technology Directorate, Department of Homeland Security; Research Fund for the Control of Infectious Disease of the Government of the Hong Kong SAR.

### **Competing Interests**

All the authors declare that no competing interests exist.

## Supplementary Material

Taiwan Influenza Epidemic Simulations **Movie 1.** Simulation of Epidemic in Taiwan seeded either in Taipei (left) or Chuanghau (right).
